# Steatosarcopenia: A New Terminology for Clinical Conditions Related to Body Composition Classification

**DOI:** 10.3390/life14111383

**Published:** 2024-10-28

**Authors:** Glaycon Michels, Guido Mattos Rosa, Guilherme Renke, Bernardo Starling-Soares

**Affiliations:** 1 IGM—Instituto Dr. Glaycon Michels, Florianópolis 88034-050, SC, Brazil; 2 Nutrindo Ideais Performance and Nutrition Research Center, Rio de Janeiro 22411-040, ES, Brazil; renke@renke.com.br; 3 Programa de Bioquímica e Imunologia, Instituto de Ciências Biológicas, Universidade Federal de Minas Gerais, Belo Horizonte 31310-250, MG, Brazil; 4 Extreme Sports Nutrition Institute—INEE, Belo Horizonte 31310-370, MG, Brazil

**Keywords:** muscle mass, sarcopenia, sarcopenic obesity, myosteatosis intramyocellular, myosteatosis intermuscular

## Abstract

Body composition analysis focuses on measuring skeletal muscle mass and total body fat. The loss of muscle function and mass is related to clinical conditions such as frailty, increased risk of falls, and prolonged hospitalizations. Despite the relevance of the definition of sarcopenic obesity, there is still a gap in the monitoring of patients who have the combination of sarcopenia and myosteatosis, regardless of the presence of obesity. Therefore, we propose a new nomenclature, steatosarcopenia, a condition characterized by the loss of mass or skeletal muscle strength and performance associated with the excessive deposition of ectopic reserve fat in muscle tissue, in the same individual, not necessarily related to excess fat total body mass. A greater understanding of this condition may assist in developing strategies for preventing and treating metabolic diseases.

## 1. Introduction

Of great interest to doctors, nutritionists, and physical educators, research publications and studies involving body composition analysis have gained increasing relevance in health sciences [1], especially with the evolution and applicability of increasingly precise non-invasive methods [2]. Notably, a substantial focus within these studies pertains to assessing fat and adipose tissue, driven by the understanding that surplus body fat correlates with heightened morbidity and mortality rates [3,4,5,6,7]. Moreover, variations in body fat distribution are observed across diverse demographic factors such as age, gender, ethnicity, and individual characteristics [8].

Skeletal muscle mass analysis has been growing, qualitatively and quantitatively (e.g., Dual-energy X-ray Absorptiometry (DEXA), computed tomography (CT), magnetic resonance imaging (MRI), bioelectrical impedance analysis (BIA)), with an increasing number of specific studies over the past few decades [1,2]. A more precise understanding of the relationship between loss of muscle function and muscle mass in clinical conditions such as frailty, increased risk of falls, prolonged hospitalizations, and other related situations is now stated [9]. In addition to these consequences of muscle mass loss, more detailed analyses have revealed that loss of muscle function, even without loss of muscle mass, namely dynapenia—an initial characteristic of sarcopenia—is associated with an increased occurrence of Alzheimer’s dementia and a decline in cognitive ability [10]. Interestingly, some authors have linked a condition termed secondary sarcopenia, which is distinct from primary sarcopenia as it is not caused by aging itself, with several factors, one of them being neurodegenerative conditions [11].

One of the significant challenges in assessing obesity and body composition lies in the selected methods, which can range from calculating Body Mass Index (BMI: *body weight (kg)/height (m)^2^*)—a simple and inexpensive method used to define overweight and obesity by the World Health Organization [12] [Global BMI Mortality Collaboration 2016]—to advanced imaging techniques such as DEXA, CT, and MRI [1]. Today, these imaging examinations are considered the gold standard methods for body composition assessment, with a preference for DEXA due to its high degree of accuracy, safety, and relatively low radiation emission [1,11]. Additionally, bioelectrical impedance analysis (BIA) is gaining relevance due to its ease and speed of use, inter-evaluator precision, and, in some models, the capacity to examine without professional interaction (Table 1) [1,13].

Adipose and skeletal muscle are important tissues in human metabolism, and they highly impact each other. Furthermore, they impact other crucial body systems—especially endocrine, nervous, and cardiovascular systems—through signaling circulatory molecules named cytokines (e.g., interleukins and myokines) [14,15]. The proportion and distribution of adipose and skeletal muscle tissues are important in various physiological, pathological, and clinical processes as these two compartments are closely linked to metabolic pathways. Adipose and skeletal muscle tissues have received significant attention regarding their endocrinological influence as they are associated with several endocrinometabolic conditions, such as type 2 diabetes mellitus—a circumstance where myosteatosis can occur independently of obesity [16,17,18]. Furthermore, sarcopenia—the combination of low muscle mass and poor muscle function [18]—has been linked to Alzheimer’s, while dyslipidemia [19] and a sedentary lifestyle [20] affect both obesity and sarcopenia. Loss of general mobility [21] and increased risk of mortality [22] are also concerns associated with these compartments, namely muscular and adipose tissues.

When it comes to the direct relationship between muscle and fat, the terms myosteatosis and sarcopenic obesity arise and are widely recognized and used in the medical literature to describe specific conditions. Myosteatosis refers to the accumulation of intra and/or intermuscular fat [23], while sarcopenic obesity describes the coexistence of obesity with loss of muscle mass and/or function [24]. Although these terms are helpful and sufficient to evaluate certain conditions, there is a significant gap in the literature regarding the approach to sarcopenia associated with myosteatosis in the same individual.

This combination of sarcopenia and myosteatosis conditions, which we propose here to denominate steatosarcopenia, hypothetically, would be linked to a worse prognosis in several health conditions already mentioned previously regarding the different systems of the body. The coexistence of these two conditions can exacerbate the effects of each, resulting in more significant loss of mobility, increased risk of falls and fractures, and a higher incidence of metabolic and cardiovascular comorbidities [18,25].

Furthermore, the identification and recognition of steatosarcopenia as a clinical condition distinct from the current ones are crucial for developing more effective diagnostic and intervention strategies. This includes the need for assessment methods that can accurately detect the simultaneous presence of muscle mass loss [26] and intramuscular fat accumulation; the definition of clear and objective concepts that can be tested and replicated in medical guidelines; and therapeutic approaches that can mitigate the adverse effects of this combined condition [27].

Therefore, this article aims to fill this gap in the literature by proposing the inclusion of the term steatosarcopenia and discussing its clinical and prognostic implications. By doing so, we hope to contribute to a better understanding and management of this complex condition, promote a more efficient approach to improving the health and quality of life of affected patients, and assist in the planning of public policies [28,29].

## 2. Research Methodology

This research was conducted independently by the four authors using the electronic databases Medline (via PubMed), ScienceDirect, and Cochrane reviews, focusing on publications dated between 1990 and May 2024. Initially, studies were selected based on their titles and abstracts. In the second stage, the selected studies were read in their entirety. The keywords used were the following: obesity OR sarcopenia OR myosteatosis, OR sarcopenic obesity OR muscle fat OR steatosarcopenia (no results found).

## 3. Currently Terminology

In the current literature, three different terms describe related alterations between muscle mass and fat deposition: *sarcopenia* [30]*—with its definitions still being updated—, myosteatosis* [17]*, and sarcopenic obesity* [31] (Table 2). These terms have gained increasing relevance in numerous fields of health sciences in recent years, given the magnitude of results obtained from correlations of these conditions with other diseases, such as the association between sarcopenia and worsened prognosis in the postoperative period of oncological diseases [32,33,34] and in chronic kidney disease [35]; the association between myosteatosis and worsened prognosis of liver diseases [23], increased insulin resistance, and type 2 diabetes mellitus [36]; and the association between sarcopenic obesity and type 2 diabetes mellitus and metabolic syndrome [11,27] and cardiovascular risk factors [24,37].

### 3.1. Myoesteatosis

The current definition of myosteatosis, as cited by Miljkovic and Zmuda, is merely the following: “ectopic fat infiltrated in skeletal muscle tissue”. According to the author, this definition is subdivided into two distinct categories: intramyocytic myosteatosis, which refers to ectopic fat deposition within myocytes in the form of lipid droplets [17]—important to note that this process often occurs to a certain extent in physiologically normal situations [38]—and intermuscular myosteatosis, which refers to visible fat deposition within the fascia surrounding the muscle [17].

### 3.2. Sarcopenia and Sarcopenic Obesity

The coexistence of obesity and sarcopenia in the same individual generally, but not necessarily, occurs in individuals older than 65 years [31]. A significant limitation in evaluating this condition arises from the fact that neither sarcopenia nor obesity currently has a unique definition. Various definitions have been proposed over the years: the term sarcopenia was first introduced by Irwin H. Rosenberg in 1988 at a convention in Albuquerque, New Mexico, to describe, simplistically, “an important change in body composition” [39]: a loss of muscle mass and function that could be related to age, disease, or normal aging. The first robust (population of 883 subjects) and widely used definition of sarcopenia was published by Baumgartner et al. ten years later in 1998, which characterized sarcopenia as an appendicular skeletal muscle mass (kg)/height^2^ (m)^2^ less than two standard deviations below the mean of a reference group of young adults [29]. In 2003, Newman and colleagues proposed an alternative definition of sarcopenia adjusted for body fat mass and height, using the data from the Aging and Body Composition Study (Health ABC Study), to appropriately adjust the prevalence of Sarcopenia in women and individuals with overweight or obesity [40]. This longitudinal observational study analyzed a group of men and women aged 70 to 79 years who did not report difficulties in daily activities (such as climbing 10 stairs or walking 400 m). For the definition of sarcopenia, a normal range of appendicular skeletal muscle mass from a group of young adults was arbitrarily chosen, and the 20th percentile was set as the comparison parameter with the elderly group using DEXA to assess body composition [40].

Since then, other definitions have been proposed to emphasize the importance of assessing physical performance and muscle strength in addition to just muscle mass indices. Various working groups have been established to improve definitions, implications, and approaches related to sarcopenia. Examples include the Asian Working Group for Sarcopenia [41,42] and the International Working Group on Sarcopenia [43]. Starting in 2010 and updated in 2019, a task force known as the European Working Group on Sarcopenia in Older People (EWGSOP [28] and EWGSOP-2 [18], respectively) became the most widely referenced source for the definition and diagnosis of sarcopenia. In 2010, the prevailing definition used a combination of low muscle mass, low muscle strength, or low physical performance, clarifying that muscle strength does not solely depend on muscle mass, and the relationship between strength and muscle mass is not linear [28]. With the revision by EWGSOP2 in 2018, the definition uses low muscle strength as the primary parameter, indicating a probable diagnosis of sarcopenia when present. The diagnosis is confirmed when there is low muscle strength and a low muscle quality or quantity [18]. When an individual exhibits low muscle strength, low muscle quantity or quality, and low physical performance, it is considered severe sarcopenic [18].

The condition described as obesity is defined as an excess of body fat that increases the risk of adverse medical conditions and mortality [44]. There is no consensus on the cut-off points for defining obesity, and its diagnosis is also related to various other metabolic and hormonal alterations [45]. The American Association of Clinical Endocrinology (AACE) recommends using body fat limits established by the World Health Organization for the diagnosis of obesity: according to DEXA parameters, increased body fat of >25% for men and >35% for women. Although, Baumgartner defines obesity body fat limits of >27% for men and >38% for women using DEXA and an equation for predicting DEXA from anthropometric data (31). Additionally, BMI > 30 kg/m^2^ and waist circumference (WC > 102 cm for men and WC > 88 cm for women) are used as indicators of visceral fat [46].

## 4. Current Terminology Limitations

Myosteatosis and sarcopenic obesity are terms that, despite addressing conditions involving alterations in muscle and adipose tissue, do not simultaneously classify them, except in obese individuals (in the case of the latter). Both conditions are widely used in studies correlating favorable outcomes in preoperative conditions, prognosis in patients with neoplasms, and patients with chronic kidney injury on dialysis, among others [32,33,34,35]. Therefore, the vast importance and technical application of these analyses are evident.

Current evidence, however, remains insufficient to evaluate health outcomes or worsening clinical conditions in individuals presenting both sarcopenia and fat deposition in muscle tissue, as this condition has yet to be firmly conceptualized in the literature. Thus, future research must focus on better understanding how this dual condition affects clinical outcomes to develop more effective and personalized intervention strategies for these patients and minimize public health expenditures resulting from complications of such conditions.

The concepts of sarcopenia and obesity lack a clear and unified definition in their widespread use. Consequently, it can be inferred that the number of individuals classified as having sarcopenic obesity is underestimated. The definition of obesity using BMI, while applicable, has proven insufficient as it does not account for body composition parameters [2]. The definition based on body fat percentage has two significant limitations: first, it ideally requires a DEXA scan [1,2], which may only sometimes be readily available depending on the patient’s location. Second, the foundational data for these definition parameters are quite flawed, stemming from inadequate bibliographic citations perpetuated over the years [8,13].

## 5. New Terminology Proposal: Steatosarcopenia

### 5.1. Steatosarcopenia Concept

**Figure 1:**
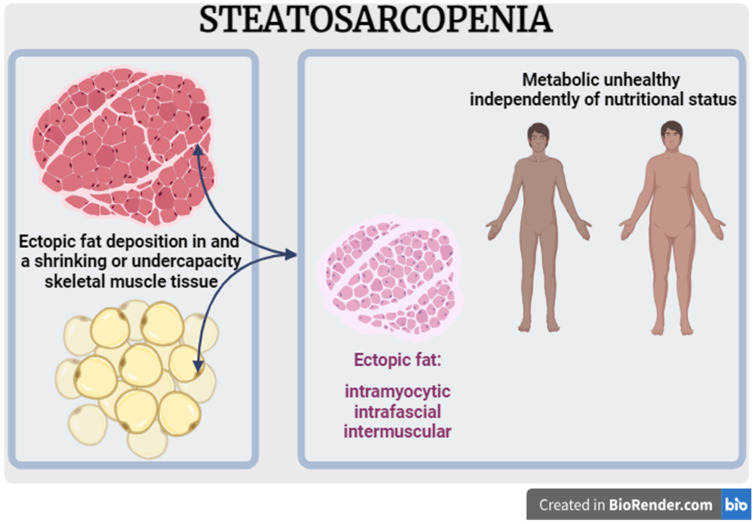
Steatosarcopenia model. Created in BioRender.com.

The definition of steatosarcopenia is the skeletal muscle mass loss or the decline of its strength and performance associated concomitantly with the excessive deposition of ectopic fat in muscle tissue in the same individual and not necessarily related to excess total body mass. In other words, it is a combination of two disorders related to skeletal muscle mass—sarcopenia and myosteatosis—coexisting in the same individual, regardless of the presence of obesity (Figure 1).

### 5.2. Clinical Relevance and Use

Considering the close correlation of the proposed condition of steatosarcopenia with the already known conditions of sarcopenic obesity and myosteatosis, it can be safely stated that diseases related to them would also be present in patients with steatosarcopenia probably on a larger scale (Table 3). According to a study published in 2004, the estimated direct cost generated by sarcopenia health care in the United States of America in 2000 was USD 18.5 billion, equivalent to USD 860 and USD 933 per sarcopenic adult man and woman, respectively [47]. Regarding costs in Brazil, a study published in 2020 presenting obesity expenses in the Unified Health System (SUS) estimated a cost reaching BRL 1.42 billion in 2018 [48]. Sarcopenic obesity studies demonstrate a variable increase in risk factors according to data from the observed literature, but it is consistently found to be positively correlated with risk of falls and postural instability [49], dyslipidemia [50], insulin resistance and hypertriglyceridemia [37], diabetes mellitus [51], depression [52], neoplasms [53], and arterial calcification [54].

In the scope of myosteatosis, which will not necessarily be associated with sarcopenia and can be affected by different clinical sessions [55], the stages also worsen in patients with average muscle mass. Studies presented a positive correlation between myosteatosis and all-cause mortality and type 2 diabetes mellitus [17], among other conditions.

## 6. Physiological Aspects

Considering that the occurrence of the condition of steatosarcopenia arises from the occurrence of the two previously mentioned conditions—myosteatosis and sarcopenia—the physiological bases will also be rooted in their origins. The pathogenesis of sarcopenia and myosteatosis is not yet fully understood. Some authors consider both processes inherent and would naturally occur with aging. However, given the pathological consequences of these conditions, it is worth considering the possibility of pathological aging, previously regarded as physiological. A comprehensive understanding of the mechanisms involved is expected to aid a better experience and development of strategies for the prevention and treatment of diseases such as obesity, type 2 diabetes mellitus, dyslipidemia, and others known to be linked to sarcopenia and myosteatosis [56,57,58].

Aging itself, along with its inherent hormonal changes in testosterone, estrogen, Growth Hormone (GH), IGF-1 (somatomedin C) levels [59], and vitamin D [60], is among the many mechanisms related to sarcopenia. The same hormonal changes are also linked to the processes of liporeplacement in skeletal muscle tissue in the case of myosteatosis. Other studies also cite the role of pro-inflammatory cytokines and oxidative stress, promoting an inflammatory state that generates sarcopenia [60,61]. Mechanistically, interleukin-1, interleukin-6, C-reactive protein, and tumor necrosis factor alpha can induce a reduction on satellite muscle cells, trigger alterations in the ubiquitin–proteasome system and negatively affect mitochondrial function via the effect of reactive oxygen species, ultimately leading to sarcopenia [60]. Interestingly, adequate levels of vitamin D have been associated with low levels of oxidative stress and pro-inflammatory cytokines [62].

## 7. Limitations

The lack of a unified definition for both sarcopenia and obesity significantly encumbers the health professional task. The condition of myosteatosis also needs a unified and widely used definition despite recent efforts to identify an optimal parameter for its definition. One of the definitions of obesity utilizing body fat percentage via DEXA is particularly weak, as it stems from a bibliographic citation inaccuracy: the original parameters used as cut-off points for defining obesity come from a study published in 1992 by Bjorntorp [63], which analyzes changes in body composition within a population of 200 individuals aged 45–78 years old. However, the study’s design and objective were not aimed at establishing obesity concepts and they even stated that the same BMI of 30 in Dutch men and women implies a 10% difference in total body fat content from a young adult when compared to a 60-year-old subject. In 1995, the World Health Organization technical report series number 854 [64] brought the data from Bjorntorp in the section “Adults 60 years of age and older” within other studies’ data. In 1998, Deurenberg [65] used the data mentioned by Bjorntorp as obesity indices, and subsequent publications perpetuated this possible mistake. It is also important to note that the study of Bjorntorp used the method of hydrostatic weighing to determine body fat mass, not the DEXA method, which composes Deurenberg data. Therefore, the definition of obesity by body fat percentage requires revisions and studies to confirm or correct the previously used cut-off points.

Considering that the definition of steatosarcopenia intimately depends on the diagnosis of myosteatosis and sarcopenia in the same individual, the same limitations apply to the proposed condition.

The present study here did not conduct a systematic review but rather a narrative review with the purpose of presenting a new nomenclature and classification aiming to assist in the development of strategies for preventing and treating the condition.

## 8. Conclusions

Despite the evolution and relevance of the definitions of sarcopenic obesity, sarcopenia, and myosteatosis, there is still a gap in the prognosis of patients who have a combination of the two disorders related to skeletal muscle mass, sarcopenia, and myosteatosis, coexisting in the same individual, regardless of the presence of obesity. We propose a new definition of steatosarcopenia characterized by the loss of mass, or skeletal muscle strength and performance, associated with the excessive deposition of ectopic, reserve fat in muscle tissue in the same individual, not necessarily associated with excess total body mass. Given the magnitude of results obtained from correlations of such conditions with other metabolic diseases, we suggest that this new terminology may assist in a better understanding and development of strategies for the prevention and treatment of diseases such as obesity, type 2 diabetes mellitus, and dyslipidemia, among others, known to be linked with the reduction in strength and skeletal muscle mass regardless of the presence of obesity. Future research may clarify prevalence and direct efforts and suggest specific treatments for this condition.

## Figures and Tables

**Table 1:** Current methods description. Data adapted from Kuriyan, 2018 [1].

Method	Advantages	Limitations	Applicability for Steatosarcopenia
BMI	Simple and inexpensive	Does not differentiate lean mass from fat mass	Limited. Does not specifically detect steatosarcopenia
DEXA	High precision, safety, low radiation	Moderate cost, not portable	Good for assessing muscle mass and body fat content
CT	High precision, evaluates intramuscular fat	High radiation, high cost	Excellent for assessing myosteatosis and muscle mass
MRI	High precision, no radiation	Expensive, not portable	Excellent for assessing myosteatosis and muscle mass
BIA	Fast, portable	Less precise than imagines methods above	Moderate, can estimate muscle mass and total fat

BMI: Body Mass Index [Body weight (kg)/height^2^ (m^2^)]; DEXA: Dual-Energy X-ray Absorptiometry; CT: Computed Tomography; MRI: Magnetic Resonance Imaging; BIA: Bioelectrical Impedance Analysis.

**Table 2:** Definition of the current denominations.

Denomination	Definition	Inclusion Parameters	References
Sarcopenia (definition updating)	A combination of low muscle mass and poor muscle function	GP: M < 27 kg W < 16 kg CST: >15 s ASM: M < 20 kg W < 15 kg ASM/height^2^: M < 7.0 kg/m^2^ W < 5.5 kg/m^2^ GS: ≤0.8 m/s SPPB: ≤8 point score TUG: ≥20 s 400 m WT: non-completion or ≥6 min	Cruz-Jentoft 2019 [18]
Myosteatosis (general)	The infiltration of fat in skeletal muscle	Despite the existence of CT, MRS, and MRI cut-off values for several disease states, there is no established consensus values for general condition.	Miljkovic 2010 [17]
Myosteatosis Intramyocellular	Intramyocytic ectopic fat deposition		Miljkovic 2010 [17]
Myosteatosis Intermuscular	Visible fat deposition within the fascia surrounding the muscle		Miljkovic 2010 [17]
Sarcopenic Obesity	Coexistence of obesity and sarcopenia in the same individual	M: ASM/height^2^: <7.26 kg/m^2^ AND BF: >27% W: ASM/height^2^: <5.45 kg/m^2^ AND BF: >38%	Baumgartner 2000 [31]

GP: grip strength; M: men; W: women; CST: chair stand test; ASM: appendicular skeletal muscle mass; GS: gait speed; SPPB: short physical performance battery; TUG: timed-up-and-go test; WT: walk test; CT: computed tomography; MRI: magnetic resonance imaging; MRS magnetic resonance spectroscopy; BF: body fat.

**Table 3:** Advantages of the establishment of the steatosarcopenia condition in various metabolic diseases.

Metabolic Disease	Advantage of Using ‘Steatosarcopenia’	Potential Impact on Treatment Strategies
Type 2 Diabetes Mellitus	Recognizes the coexistence of muscle loss and fat infiltration, which can occur independently of obesity	More comprehensive approach to glucose regulation Combined focus on muscle preservation and intramuscular fat reduction Tailored exercise and nutritional interventions
Dyslipidemia	Addresses both muscle quality and quantity issues that contribute to lipid metabolism	Targeted therapies to improve muscle metabolism and reduce ectopic fat Personalized lipid-lowering strategies considering muscle health
Metabolic Syndrome	Captures the interplay between muscle health and fat distribution more accurately	Holistic interventions addressing both muscle and fat compartments Enhanced risk stratification for cardiovascular complications
Non-alcoholic Fatty Liver Disease (NAFLD)	Recognizes the role of both muscle loss and ectopic fat accumulation in liver health	Combined strategies to improve muscle mass and reduce overall and ectopic fat Liver-specific interventions considering muscle–liver axis
Osteoporosis	Acknowledges the relationship between muscle health, fat infiltration, and bone metabolism	Integrated approach to improve bone density, muscle mass, and reduce fat infiltration Fall prevention strategies considering both muscle strength and quality
Cardiovascular Diseases	Provides a more complete picture of body composition changes affecting cardiovascular risk	Comprehensive cardiac rehabilitation programs Targeted interventions to improve muscle function and reduce cardiovascular risk factors

## Data Availability

Data are contained within the article.
